# Circulating plasma miR-23b-3p as a biomarker target for idiopathic Parkinson's disease: comparison with small extracellular vesicle miRNA

**DOI:** 10.3389/fnins.2023.1174951

**Published:** 2023-11-15

**Authors:** Sanskriti Rai, Prahalad Singh Bharti, Rishabh Singh, Simran Rastogi, Komal Rani, Vaibhav Sharma, Priya Kumari Gorai, Neerja Rani, Bhupendra Kumar Verma, Thota Jagadeshwar Reddy, Gyan Prakash Modi, Krishna Kishore Inampudi, Hem Chandra Pandey, Sanjay Yadav, Roopa Rajan, Fredrik Nikolajeff, Saroj Kumar

**Affiliations:** ^1^Department of Biophysics, All India Institute of Medical Sciences, New Delhi, India; ^2^Department of Pathology and Laboratory Medicine, All India Institute of Medical Sciences Bibinagar, Hyderabad, India; ^3^Department of Health, Education and Technology, Luleå University of Technology, Luleå, Sweden; ^4^Department of Anatomy, All India Institute of Medical Sciences, New Delhi, India; ^5^Department of Biotechnology, All India Institute of Medical Sciences, New Delhi, India; ^6^Analytical Department, CSIR-Indian Institute of Chemical Technology, Hyderabad, India; ^7^Department of Pharmaceutical Engineering and Technology, Indian Institute of Technology BHU, Varanasi, India; ^8^Department of Transfusion Medicine, All India Institute of Medical Sciences, New Delhi, India; ^9^Department of Biochemistry, All India Institute of Medical Sciences Raebareli, Uttar Pradesh, India; ^10^Department of Neurology, All India Institute of Medical Sciences, New Delhi, India

**Keywords:** Parkinsion's disease (PD), small extracellular vesicle (sEV), miRNA—microRNA, miR-23b-3p, biomarker

## Abstract

**Background:**

Parkinson's disease (PD) is an increasingly common neurodegenerative condition, which causes movement dysfunction and a broad range of non-motor symptoms. There is no molecular or biochemical diagnosis test for PD. The miRNAs are a class of small non-coding RNAs and are extensively studied owing to their altered expression in pathological states and facile harvesting and analysis techniques.

**Methods:**

A total of 48 samples (16 each of PD, aged-matched, and young controls) were recruited. The small extracellular vesicles (sEVs) were isolated and validated using Western blot, transmission electron microscope, and nanoparticle tracking analysis. Small RNA isolation, library preparation, and small RNA sequencing followed by differential expression and targeted prediction of miRNA were performed. The real-time PCR was performed with the targeted miRNA on PD, age-matched, and young healthy control of plasma and plasma-derived sEVs to demonstrate their potential as a diagnostic biomarker.

**Results:**

In RNA sequencing, we identified 14.89% upregulated (fold change 1.11 to 11.04, *p* < 0.05) and 16.54% downregulated (fold change −1.04 to −7.28, *p* < 0.05) miRNAs in PD and controls. Four differentially expressed miRNAs (miR-23b-3p, miR-29a-3p, miR-19b-3p, and miR-150-3p) were selected. The expression of miR-23b-3p was “upregulated” (*p* = 0.002) in plasma, whereas “downregulated” (*p* = 0.0284) in plasma-derived sEVs in PD than age-matched controls. The ROC analysis of miR-23b-3p revealed better AUC values in plasma (AUC = 0.8086, *p* = 0.0029) and plasma-derived sEVs (AUC = 0.7278, *p* = 0.0483) of PD and age-matched controls.

**Conclusion:**

We observed an opposite expression profile of miR-23b-3p in PD and age-matched healthy control in plasma and plasma-derived sEV fractions, where the expression of miR-23b-3p is increased in PD plasma while decreased in plasma-derived sEV fractions. We further observed the different miR-23b-3p expression profiles in young and age-matched healthy control.

## Introduction

Idiopathic Parkinson's disease is the second most common progressive neurodegenerative disorder after Alzheimer's disease (AD) (Kalia and Lang, 2015). According to the Dorsey et al. (2018), the worldwide burden of PD has more than doubled over the past two decades from 2.5 million patients in 1990 to 6.1 million patients in 2016 (Dorsey et al., 2018). The typical motor features of PD include bradykinesia, resting tremor, rigidity, and postural instability, and these symptoms worsen over time. The hallmark pathological feature in PD is the profound degeneration of the dopaminergic neurons in the substantia nigra pars compacta (SNpc) and the intraneuronal inclusion of Lewy bodies (Shults, 2006; Dickson, 2012). Loss of dopaminergic neurons, which projects widely within the striatum and pallidum, results in a state of low dopamine (Fearnley and Lees, 1991). As a result of this, there is a denervation of the nigrostriatal pathway that ultimately leads to diminished dopamine levels in the striatum, which is responsible for the appearance of the cardinal motor symptoms in PD (Kordower et al., 2013). A major conundrum in Parkinson's disease is its striking clinical variability, where idiopathic Parkinson's disease features an asymmetrical condition with a good levodopa response (Hughes et al., 1992). The clinical manifestations are unilateral in early disease and encompass a spectrum from a tremor-dominant (TD) phenotype to a postural instability gait difficulty (PIGD) phenotype with rapid progression accompanied by rigidity, severe gait dysfunction, falls, and faster cognitive decline (Aleksovski et al., 2018). Therefore, there is a diagnostic heterogeneity which is a well-recognized hurdle in the development of new therapeutic strategies for Parkinson's disease.

Microscopically, the defining feature of Parkinson's disease (PD) is the presence of abnormal protein aggregates known as Lewy bodies (LBs) within cell bodies of neurons (Spillantini et al., 1998; Kim et al., 2014). LBs are inclusion bodies that are composed of a granular and fibrillar core containing a number of proteins, including ubiquitin, tau, parkin, heat shock proteins (HSPs), oxidized/nitrated proteins, cytoskeletal proteins such as neurofilaments, MAPs, and tubulin, and proteasomal and lysosomal elements (Xia et al., 2008; Goedert et al., 2013). LBs are surrounded by a halo that is primarily composed of α-synuclein. α-synuclein is ubiquitously expressed in the body, and under normal circumstances, it folds into an α-helical structure through the N-terminal when it interacts with negatively charged lipids, such as the phospholipids that make up cell membranes (Eliezer et al., 2001; Bartels et al., 2011). However, in PD and other synucleinopathies, it acquires a β-sheet-rich amyloid-like filamentous structure that is prone to aggregate and becomes abnormally phosphorylated and aggregated (George, 2001; Fujiwara et al., 2002). These misfolded α-synuclein filaments are found within LBs and are 5–10 nm long (Fujiwara et al., 2002). Conformational changes that lead to abnormal α-synuclein aggregation are serine 129 phosphorylation, ubiquitination, and C-terminal truncation (Samuel et al., 2016; Zhang et al., 2019a). Different species of α-synuclein are found in the PD brain, including unfolded monomers, soluble oligomers, protofibrils, and high molecular weight insoluble fibrils (Baba et al., 1998). The early oligomeric forms have been documented to be more toxic in various studies (Danzer et al., 2009). The majority of PD cases reported are sporadic, i.e., only ~10% of patients have a positive family history (Klein and Westenberger, 2012). There are six genes unequivocally linked to heritable, monogenic PD, which includes mutations in SNCA (PARK1 = 4) and LRRK2 (PARK8) that are responsible for autosomal dominant PD forms, while mutations in Parkin (PARK2), PINK1 (PARK6), DJ-1 (PARK7), and ATP13A2 (PARK9) are accountable for PD with an autosomal recessive (AR) mode of inheritance (Thomas and Beal, 2007; Kalia and Lang, 2015; Day and Mullin, 2021).

Currently, Parkinson's disease has a clinical diagnosis, and the accuracy of which relies on a thorough clinical examination and neuroimaging techniques. There is no objective molecular or biochemical test for PD. Cerebrospinal fluid (CSF) is considered to be ideal to interrogate biomarkers for neurodegenerative diseases such as PD. Cumulative data, including meta-analyses, support that a decrease in CSF α-synuclein is observed in PD (Sako et al., 2014) and that a decrease in amyloid β42, tau, and phosphorylated tau level is an indicator of cognitive decline in PD (Hu et al., 2017). Neurofilament light chain (NfL) is a structural protein highly expressed in axons and released upon neuronal damage, rendering it a robust marker for neuronal injury, where neurofilament light chains were able to discriminate PD from other neurodegenerative disorders (Bridel et al., 2019). However, CSF is not as easily accessed as other body fluids such as blood and urine, whose collection is minimally invasive compared with CSF. Increased total plasma/serum α-syn levels are observed in PD and primarily occur in the early phases of the disease (El-Agnaf et al., 2006; Tinsley et al., 2010); however, there are studies where plasma/serum concentrations of oligomeric and phosphorylated species of α-synuclein show no difference (Zubelzu et al., 2022). The source of heterogeneity for α-syn measurements could be red blood cell (RBC) contamination of other blood fractions as 99% of α-syn in human blood is present in RBCs and any transference to plasma/serum or RBC lysis could explain the huge heterogeneity of the observed results (Mollenhauer et al., 2010). The concentration of α-synuclein in plasma-derived sEV is reported to be substantially higher in PD patients, and α-syn levels in such sEVs correlated with the disease severity (Shi et al., 2014). In addition, phosphorylated tau (T181) is reported to be higher in plasma-derived sEVs and higher in Parkinson's disease than in control individuals (Blommer et al., 2023). Altered miRNA profiles have been reported in PD patients compared with control where they have reported upregulation of let-7g-3p, miR-22, miR-23a, miR-24, miR-153, miR-222, and miR-331-5p and downregulation of miR-19b-3p miR-505 in PD patients compared with control (Gui et al., 2015; Yao et al., 2018; Cai et al., 2021). MicroRNAs (miRNAs) are a class of non-coding single-stranded small RNAs that are 18–25 nucleotides long and play a major role in biological function and disease development (Bartel, 2018). Since nearly 70% of the known miRNAs are expressed in the mammalian brain, they may be valuable biomarkers for detecting early-onset neurodegenerative disorders (Hussein and Magdy, 2021). The important functional roles of miRNA in neurogenesis, neuronal differentiation, neuroprotection, survival, and pathogenesis of neurological disorders have been also extensively studied and reviewed (Cao et al., 2006; Pandey et al., 2015; Brennan and Henshall, 2020; Jauhari et al., 2020). Given the importance of miRNA in brain functional regulation, it is not surprising that dysregulation of miRNAs may contribute to the pathogenesis and pathological process of Parkinson's disease (da Silva et al., 2016; Hoss et al., 2016). The miRNA expression profile in the extracellular environment reflects a pathological state, and changes in the expression level of miRNAs can be used for differential diagnosis of diseases such as Parkinson's disease. However, there are chances that the peripheral miRNAs can easily be degraded by RNases. The circulating extracellular vesicles are liable to carry miRNAs as cargo, thus preventing their degradation by the widely present nucleases in the body fluids (Rastogi et al., 2021).

Extracellular vesicles (EVs) are delimited by bi-lipid membranes and are released by most cells (Admyre et al., 2003; Caby et al., 2005). Different classes of EVs are determined by their cellular sites of EV biogenesis, which are the plasma membrane (PM) and the endosomal system (Zhang et al., 2019b). Microvesicles, ectosome, and microparticles are used to describe vesicles that are directly shed from the PM, while the term “exosome” or “small extracellular vesicles (sEVs)” specifically refers to intraluminal vesicles (ILVs) formed in the multivesicular body (MVB) that are released from the cell by fusion of the MVB with the PM (Taylor and Gercel-Taylor, 2011). CNS-derived sEVs also have been shown to cross the blood–brain barrier in the bloodstream and therefore have drawn substantial attention as a source of biomarkers for various neurodegenerative diseases as they can be isolated *via* minimally invasive blood and report on the biochemical status of the CNS (Rastogi et al., 2021; Terstappen et al., 2021). As sEVs can freely pass through the blood–brain barrier, they reflect the condition of secreting cells, thus isolating CNS-derived sEVs from the blood is theoretically simple, and the approach has great promise (Batrakova and Kim, 2015; Hornung et al., 2020). In this study, we screened sEV-derived miRNAs that were differentially expressed in PD using small RNA-sequencing and their validation in patient samples using qPCR to demonstrate their potential as diagnostic biomarkers for PD.

## Materials and methods

### Subject recruitment and plasma separation

Blood samples were collected from PD patients (*n* = 16), respective age-matched controls (*n* = 16), and young healthy controls (*n* = 16) from All India Institute of Medical Sciences, New Delhi (Table 1, Supplementary Table 1). The clinical diagnosis was in accordance with the guidelines of the Movement Disorder Society (MDS) clinical diagnostic criteria for Parkinson's disease (Postuma et al., 2018). All subjects were recruited after obtaining a written signed consent form. The ethical clearance was obtained by the Institutional Ethics Committee of All India Institute of Medical Sciences, New Delhi, India (Ref. No.: IECPG-766/30.11.2022). The peripheral blood samples were collected in EDTA vials (BD Biosciences) using venepuncture. The upper plasma layer was separated from the blood samples by centrifugation at 1,900 g for 10 min at 4°C. Additional centrifugation of 10,000 g for 30 min was carried out to remove additional cellular debris and microvesicles (Nigro et al., 2021) and minimize contamination of cell-free nucleic acids by gDNA and RNA derived from damaged blood cells (Baranyai et al., 2015; Lobb et al., 2015; Gupta et al., 2018). The clarified plasma was frozen in aliquots at −80°C, and before usage, samples were thawed and centrifuged for 5 min at 3,000 g and 4°C.

**Table 1:** Demographic details of all subjects.

	**Young controls**	**Age-matched controls**	**PD patients**
Number	16	16	16
Age in years (mean ± SD)	25.94 ± 2.542	55.19 ± 12.65	55.63 ± 14.17
Gender (male %)	87.5%	81.25%	81.25%

### Isolation of sEVs

sEVs were isolated from the human plasma samples using the chemical precipitation method as described (Rani et al., 2021). The plasma samples were thawed and centrifuged at 10,000 g for 30 min at 4°C. The clarified plasma (360 μl) was collected, filtered through a 0.22 μm filter, and then treated with chemical precipitation (14% PEG). The sample was allowed to incubate overnight at 4°C, followed by centrifugation at 13,000 g for 1 h at 4°C. The supernatant was discarded, and the pellet was washed with 1 × PBS. Finally, 400 μl of 1 × PBS was used to resuspend the pellet. The purified fraction containing small extracellular vesicles was obtained, which was further filtered through a 100 kDa filter to obtain a final volume of 200 μl.

### Transmission electron microscopy

The ultrastructural morphology of isolated sEVs was studied by transmission electron microscopy (TEM). The isolated sEV-enriched fraction was diluted with 1 × PBS in 1:100 ratios. The diluted suspension was adsorbed on a carbon-coated copper grid (01843, Ted Pella) for 30 min at RT. The grids were blot dried and stained with 1% aqueous uranyl acetate solution for 15 s and observed under the transmission electron microscope (Talos S, Thermo Scientific, USA).

### Nanoparticle tracking analysis

Nanoparticle tracking analysis (NTA) gives information on particle size distribution and concentration in solution. The quantification of sEVs was performed using the NTA system ZetaView (Particle Matrix, Germany) in scatter mode. Purified sEVs were 1,000-fold diluted in 1 × PBS, and 0.5 ml of the sample was injected into the NTA setup for analysis. Three cycles were performed by scanning 11 cell positions each and capturing 60 frames per position (video setting: high) under the following settings: focus: autofocus; camera sensitivity for all samples: 80.0; shutter: 150; scattering intensity: 5.0; embedded laser at 488 nm; cell temperature: 25°C. The videos were captured using a CMOS camera and analyzed by the in-built ZetaView software 8.05.12 with specific analysis parameters as follows: maximum particle size: 1,000, minimum particle size 10, minimum particle brightness: 30.

### Western blot

Total protein concentration in the sEV-enriched fraction was determined by a bicinchoninic acid (BCA) protein assay kit (22802, Pierce ThermoFisher Scientific) using bovine serum albumin (BSA) as standard. All samples were normalized by the initial biofluid input volume, and the sEV-enriched sample was mixed with the sample loading dye and loaded in an equal volume of 20 μl to run on a 12% SDS–PAGE. The obtained gel was subjected to the wet mode of Western blotting using the BioRad Western blotting apparatus. The proteins were transferred from gel to 0.22 μm PVDF membrane after membrane blocking using 3% BSA in Tris-buffered saline containing 0.1% of Tween 20 (TBST). Primary antibodies (1:5,000 dilution of antibody in 1.5% BSA in TBST) of anti-CD9 (PA5-86534, Invitrogen), anti-Flotillin-1 (PA5-17127, Invitrogen), and anti-L1CAM (MA1-46045, Invitrogen) were incubated overnight at 4°C. The blot was developed by HRP-based electroluminescence using a Femto LUCENT™ PLUS-HRP kit (Gbiosciences).

### RNA isolation

**Figure 1:**
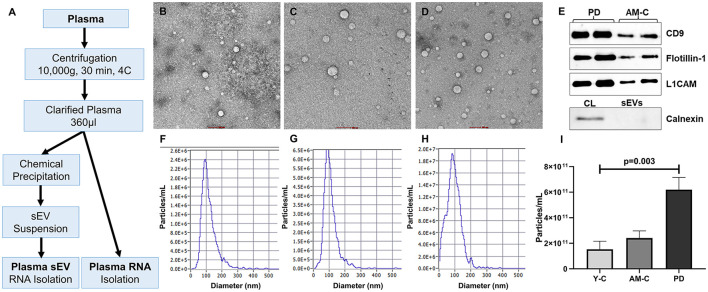
Work plan, characterization, and validation of sEVs. **(A)** The work plan of sEV-derived miRNA and plasma miRNA isolation. Morphological characterization of isolated sEV through transmission electron microscopy in young controls **(B)**, age-matched controls **(C)**, and PD patients **(D)** (scale bar 100 nm). **(E)** Western blot of sEV markers (CD9, Flotillin-1), neuronal marker (L1CAM), and a negative marker of sEVs (Calnexin) for sEV validation. Graphical representation of the distribution of the size of sEV sub-populations (nm) vs. concentration (particle/ml) in young controls **(F)**, age-matched controls **(G)**, and PD patients **(H)**. **(I)** Comparison of sEV concentration in young controls, age-matched controls, and PD patients (*p* = 0.003).

Plasma-derived small RNAs were isolated using the miRNeasy Plasma/Serum advanced isolation kit (217240, Qiagen) following the manufacturer's protocol. Total RNA from plasma-derived sEVs was isolated using a Total RNA Purification kit (17200, Norgen Biotek) following the manufacturer's protocol (Figure 1A). The initial biofluid input volume used for RNA extraction was the same for both sample types (200 μl).

### Library preparation and sequencing

Small RNA sequencing (smRNA) libraries were prepared with a QIAseq miRNA Library Kit protocol (331502, Qiagen). Overall, 5 μl of total RNA samples from young controls and PD patients was used as starting material. 3′ adapters were ligated to the specific 3′OH group of micro RNAs followed by ligation of 5′ adapter. Adapter ligated fragments were reverse transcribed with Unique Molecular Index (UMI) assignment by priming with reverse transcription primers. cDNA, thus, formed was enriched and barcoded by PCR amplification (22 cycles). The 3′ and 5′ adapters used in the preparation are 3′ AACTGTAGGCACCATCAAT and 5′ GTTCAGAGTTCTACAGTCCGACGATC. The Illumina-compatible sequencing libraries were quantified by Qubit fluorometer (Thermo Fisher Scientific, MA, USA), and fragment size distribution of the libraries was analyzed on Agilent 2200 TapeStation. The sequencing was performed on Illumina NovaSeq 6000 sequencing platform following the manufacturer's instructions.

### Data analysis

Illumina single-end sequencing was carried out for 50 cycles on the Illumina NovaSeq 6000 High Output sequencing platform following the manufacturer's instructions. The raw reads were processed for filtering specific length of 16–40 bases and were mapped to the *Homo sapiens* (GRCh38) genome; these reads were further mapped to the miRBase database (Kozomara and Griffiths-Jones, 2011). The unmapped reads which should only comprise small RNAs were used for the classification of known and novel miRNAs and target prediction. The raw data were processed by srnaworkbenchV3.0_ALPHA (Stocks et al., 2012), which was used to trim the 3′ adapter, perform length filtering (elimination of reads <16 and >40 bp), and filter contaminated reads of other ncRNAs (r-, t-, sn-, and sno-RNAs) for obtaining the final clean reads. Conserved miRNAs were identified by employing homology sequence alignment against matured humans from miRBase. Novel miRNAs were identified based on the secondary structure prediction.

### Differential expression analysis and target prediction

Read count across all known and novel miRNAs was generated by taking the count of reads aligning to a particular miRNA. This approach was used in understanding the expression pattern of miRNAs in different groups of our study. Differential expression (DE) analysis was carried out using the DESeq tool (Anders and Huber, 2010). For each miRNA with differential expression between Parkinson's disease (PD) patient and healthy control (HC), its potential target miRNAs were predicted by the miRanda tool (John et al., 2004). Next, we used Blast to align the sequences of target miRNAs with known sequences in Gene Ontology (GO) and Kyoto Encyclopedia of Genes and Genomes (KEGG) database and confirmed the potential biological functions of target miRNAs.

### Quantitative real-time PCR

For cDNA synthesis, a reverse transcription reaction was performed with the miRCURY LNA RT Kit (339340, Qiagen), as described by the manufacturer. miRCURY LNA SYBR Green PCR Kit (339346, Qiagen) and miRCURY LNA miRNA PCR Assay were used for the qRT-PCR reactions. miR-16-5p was used as endogenous controls in miRNA qRT-PCR analysis. All qRT-PCR reactions were conducted in AriaMx Real-Time PCR System (Agilent Technologies). The comparative CT method (ΔΔCt method) was used for the qRT-PCR data analysis.

### Statistical analysis

Descriptive statistical analysis was used. The qPCR data involving the comparison of controls (age-matched and young control) and Parkinson's disease patients were analyzed using GraphPad Prism 9.0 software. Statistical significance was calculated using the Mann–Whitney test. Significance was considered at *p* < 0.05. The correlation was calculated using Spearman's rank correlation. The ROC analyses were performed to determine the diagnostic accuracy. A non-parametric Kruskal–Wallis test was used for group analysis.

## Results

### Characterization and validation of sEVs

Characterization and validation of purified small extracellular vesicles (sEVs) were carried out in accordance with the minimal information for studies of extracellular vesicles (MISEV) guidelines (Théry et al., 2018). To assess the purity and yield of the small extracellular vesicle (sEV), transmission electron microscopy (TEM) and nanoparticle tracking analysis (NTA) were performed. Coomassie blue staining was also performed to check the total protein level in total plasma and plasma-derived sEV (Supplementary Figure 2). The morphological characterization and assessment of the purity of sEVs were performed through transmission electron microscopy (Figures 1B–D). The isolated sEVs are reported in the 50–90 nm range that appears as spherical vesicles with a lipid bilayer. We observed a higher sEVs population in the PD patients than in age-matched and young healthy controls. In the NTA, we observed the size and concentration distribution of sEVs in all the subjects (dilution factor 1,000 ×). Figures 1F–H shows the mean size of the sEVs and the concentrations of sEVs in particle/ml with the diameter of particles in the nanometer range (Supplementary Table 3). The mean size appeared to be ~80.15 ± 28.20 nm in young controls, 89.18 ± 14.59 nm in age-matched controls, and 92.33 ± 13.08 nm in the PD patient group. In addition, there is a significant increase in sEVs in the plasma of PD patients than the controls (*p* = 0.003; Figure 1I). Figure 1E shows the validation of the sEVs; Western blotting was performed using sEV markers CD9 (Supplementary Figure 4) and Flotillin-1 (Supplementary Figure 5) and a neuronal marker L1CAM antibodies (Supplementary Figure 6). Western blot of Calnexin (sEV-exclusion marker) was also performed where the calnexin band was present only in the cell lysate (Figure 1E, Supplementary Figure 7).

### Differential expression profile of sEVs-derived mirnas in PD patients and control

**Figure 2:**
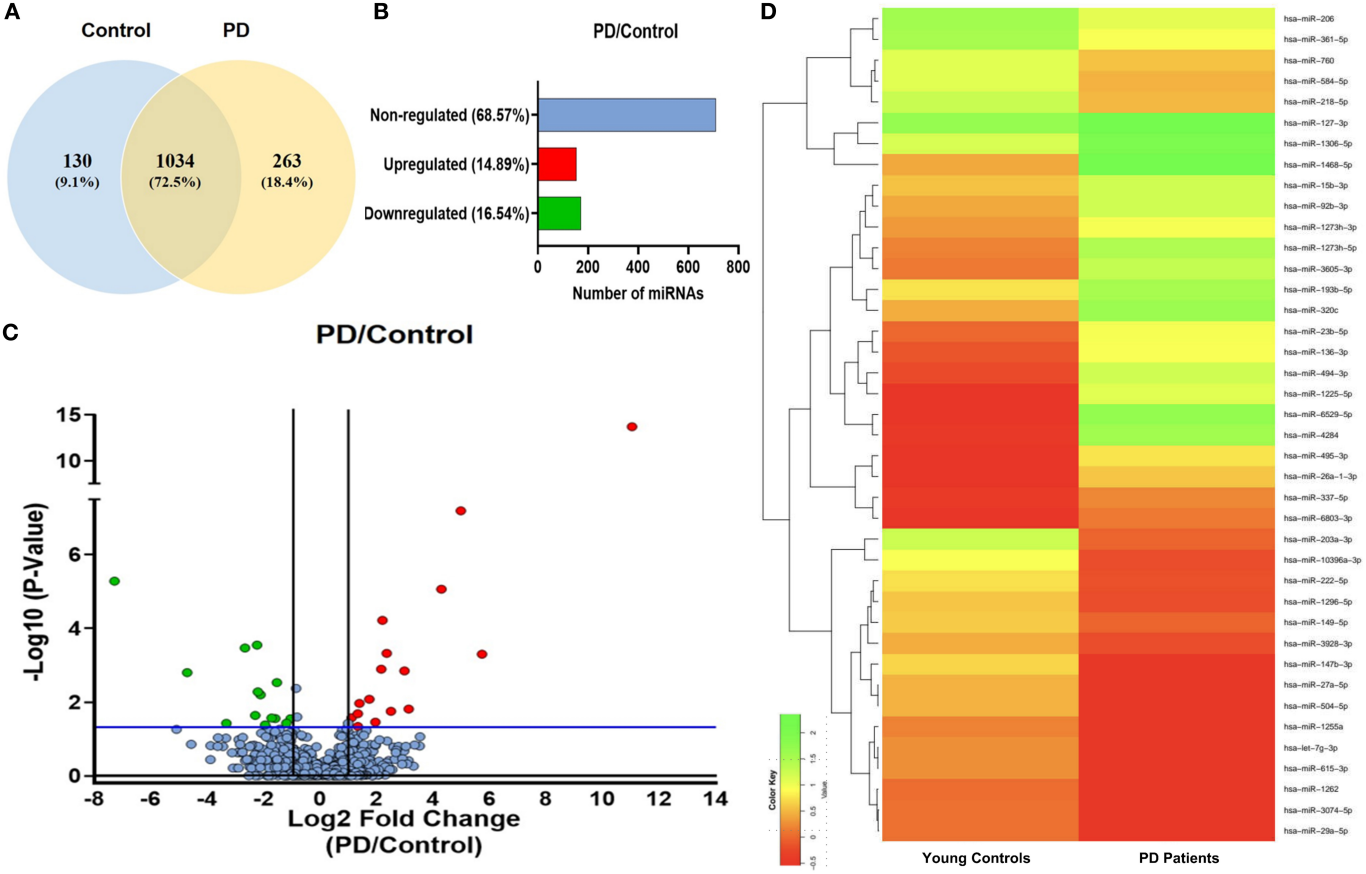
The expression profile of differentially expressed miRNAs in PD and controls. **(A)** A Venn diagram showing total miRNAs dysregulated in PD and controls. **(B)** A bar diagram showing the percentage of dysregulated miRNAs in PD and controls. **(C)** A volcano plot of differentially expressed miRNAs in PD and controls. **(D)** A heatmap showing the global profile of differentially expressed miRNA in young controls and PD patients in plasma-derived sEVs.

sEVs-derived RNA libraries were equimolar-pooled and sequenced to obtain 12–15 million single-end reads and a comprehensive profile of sEVs-derived miRNAs in PD patients and controls. The data obtained were analyzed using a standard small RNA analysis pipeline, whereby 1,427 miRNAs were identified. A total of 1,034 miRNAs were common to both the PD and control groups while 263 miRNAs were specific to the PD group and 130 to the control group, respectively (Figure 2A). Among these miRNAs, 14.89% were upregulated and 16.54% were downregulated (Figure 2B). The log_2_ fold change (Figure 2C) ranged from 11.04 to 1.115526 in the upregulated group (Table 2) while it varied from −7.28 to −1.04643 in the downregulated group (Table 3). To get a global profile of differentially expressed miRNA (DEM) in the young control and PD groups, a heatmap of the top 20 DEM in plasma-derived sEV fraction is presented in Figure 2D. Differential expression analysis of known miRNAs from control and diseased conditions was performed also on the basis of their read counts, to understand the expression patterns in our study.

**Table 2:** Upregulated miRNAs in PD.

**S. No**.	**miRNA**	**Fold change**	**Log_2_ fold change**	***p*-value**
1	hsa-miR-3074-5p	2,106.864	11.04088	2.02E-14
2	hsa-miR-1231	53.11773	5.731122	0.000502
3	hsa-miR-106b-3p	31.50452	4.977487	6.44E-08
4	hsa-miR-3184-5p	19.64099	4.295796	8.67E-06
5	hsa-miR-6858-5p	8.8	3.137504	0.015418
6	hsa-miR-23b-3p	7.912209	2.98408	0.001426
7	hsa-miR-708-3p	5.687215	2.507722	0.017674
8	hsa-miR-944	5.122277	2.356785	0.000475
9	hsa-miR-203b-5p	4.619243	2.207656	6.06E-05
10	hsa-miR-95-3p	4.485866	2.165386	0.001278
11	hsa-miR-561-5p	3.873629	1.953686	0.034862
12	hsa-miR-205-5p	3.346643	1.742715	0.008343
13	hsa-miR-224-5p	2.617928	1.388425	0.010804
14	hsa-miR-203a-3p	2.526352	1.337056	0.045801
15	hsa-miR-556-3p	2.519448	1.333108	0.02065
16	hsa-miR-450b-5p	2.166739	1.115526	0.02651

**Table 3:** Downregulated miRNAs in PD.

**S. No**.	**miRNA**	**Fold change**	**Log_2_ fold change**	***p*-value**
1	hsa-miR-106a-5p	0.006434	−7.28007	5.24E-06
2	hsa-miR-3940-3p	0.038308	−4.7062	0.001578
3	hsa-miR-4716-3p	0.099994	−3.32202	0.037687
4	hsa-miR-19b-3p	0.157704	−2.6647	0.000339
5	hsa-miR-4732-5p	0.202952	−2.30079	0.022893
6	hsa-miR-143-5p	0.212521	−2.23432	0.000283
7	hsa-miR-3610	0.21658	−2.20703	0.005246
8	hsa-miR-3180-5p	0.231643	−2.11003	0.006336
9	hsa-miR-4440	0.259708	−1.94504	0.041208
10	hsa-miR-3648	0.304168	−1.71706	0.026827
11	hsa-miR-150-3p	0.331983	−1.59082	0.027726
12	hsa-miR-625-3p	0.345623	−1.53273	0.002949
13	hsa-miR-6892-5p	0.435867	−1.19804	0.037351
14	hsa-miR-486-5p	0.484165	−1.04643	0.028155

### Target gene prediction and GO/KEGG pathway enrichment analysis

**Figure 3:**
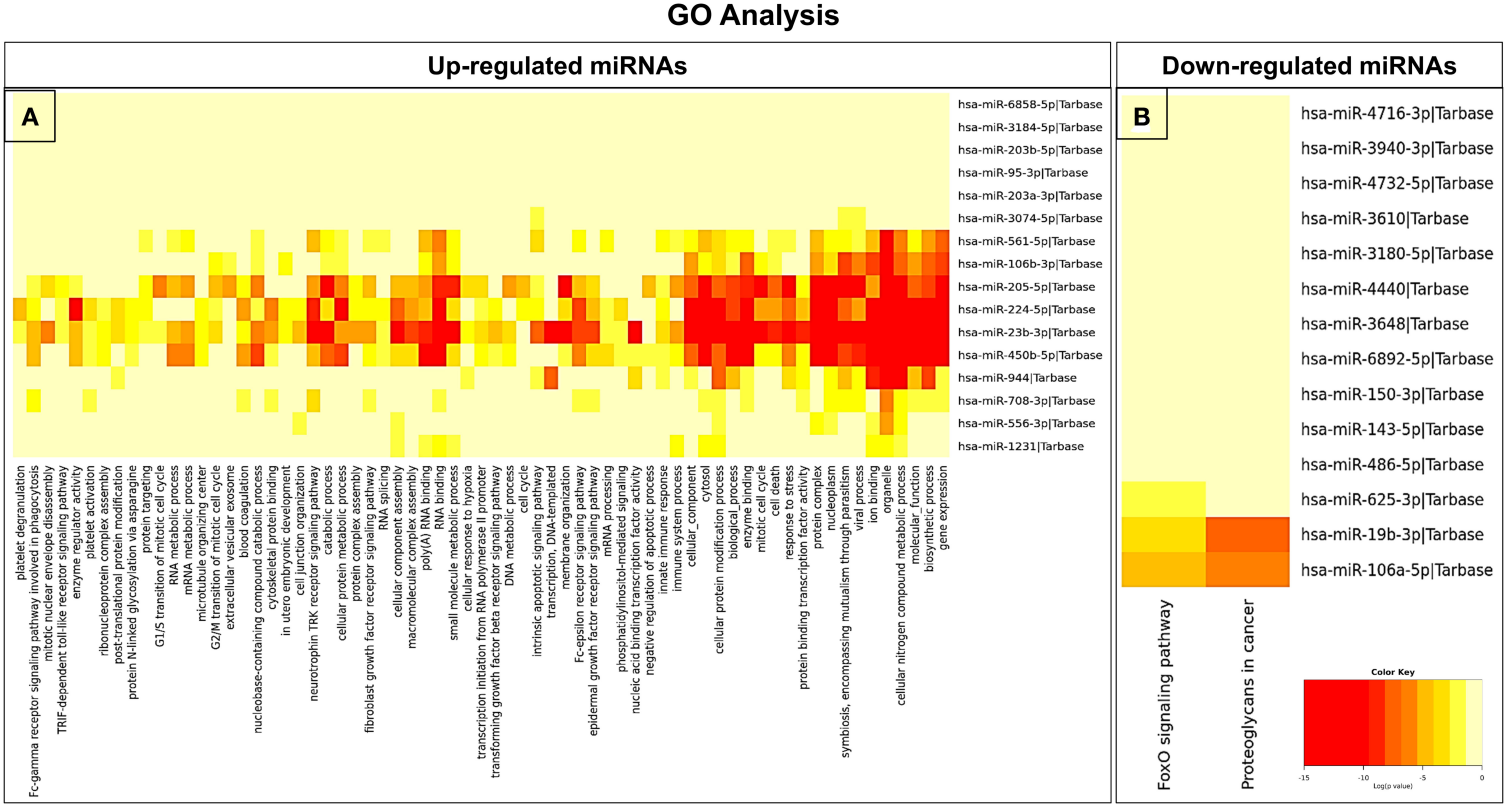
GO pathway enrichment analysis of dysregulated miRNAs. **(A)** Upregulated and **(B)** downregulated miRNAs were analyzed by the GO pathway analysis to identify related signaling pathways.

**Figure 4:**
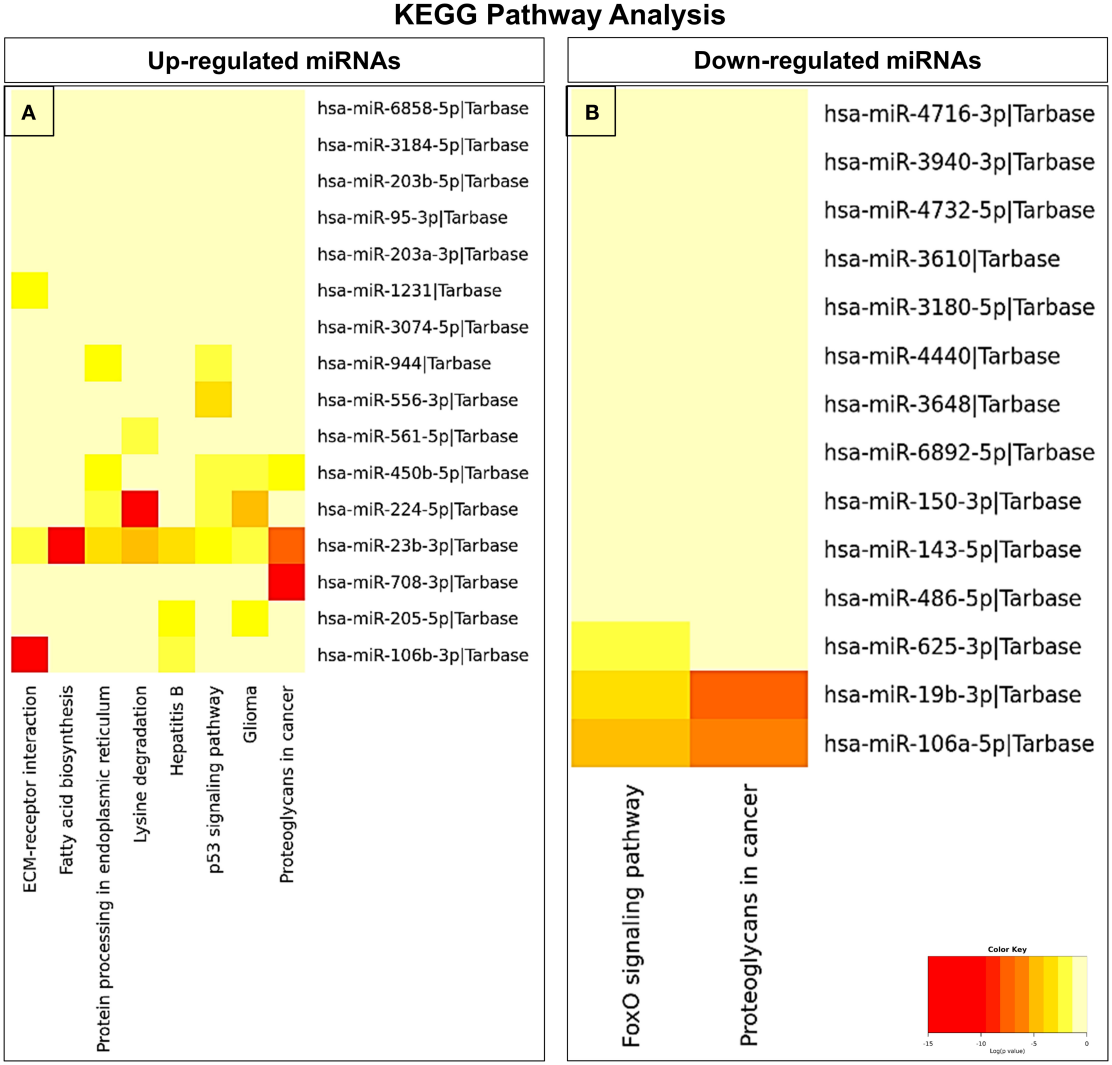
KEGG pathway enrichment analysis of dysregulated miRNAs. **(A)** Upregulated and **(B)** downregulated miRNAs were analyzed by KEGG pathway analysis to identify related signaling pathways.

In an attempt to understand the potential function of sEVs-derived differentially expressed miRNAs, we performed Gene Ontology (GO) and Kyoto Encyclopedia of Genes and Genomes (KEGG) pathway analyses. Interestingly, in our study, the functional enrichment GO analyses revealed that the sEVs-derived miRNAs-regulated target genes included innate immune response, apoptosis, and neurotrophin signaling pathway which play a pivotal role in the disease pathophysiology (Figure 3). Similarly, regulatory networks such as the TGF-beta signaling pathway, FoxO signaling pathway, cytoskeletal protein binding, cell junction organization, and cancer-associated pathways were identified (Figure 4). In addition, the regulatory roles of these sEVs-derived miRNA target genes which included transcription initiation, RNA splicing, and mRNA processing also indicate a possible role in transcriptional regulation.

### Selection of miRNA panels for qPCR validation and assessing their relative abundance in sEVs and plasma

Four miRNAs were selected based on their differential expression profile for validation through qPCR. These were also previously reported in PD literature: miR-23b-3p (Su et al., 2018; Cai et al., 2021), miR-29a-3p (Chi et al., 2018; Jiang et al., 2021; Ahmadzadeh-Darinsoo et al., 2022), and miR-19b-3p (Gui et al., 2015; Uwatoko et al., 2019) while miR-150-3p was not previously studied in PD. An equal volume of sample was used for small RNA isolation from both plasma-derived sEVs and whole plasma. cDNA was synthesized using a miRNA-specific LNA system based on universal reverse transcription (RT), which was followed by real-time PCR amplification with LNA-enhanced primers for the above-mentioned target miRNAs. However, only miR-23b-3p gave us significant results among the four miRNAs (Supplementary Figure 8).

**Figure 5:**
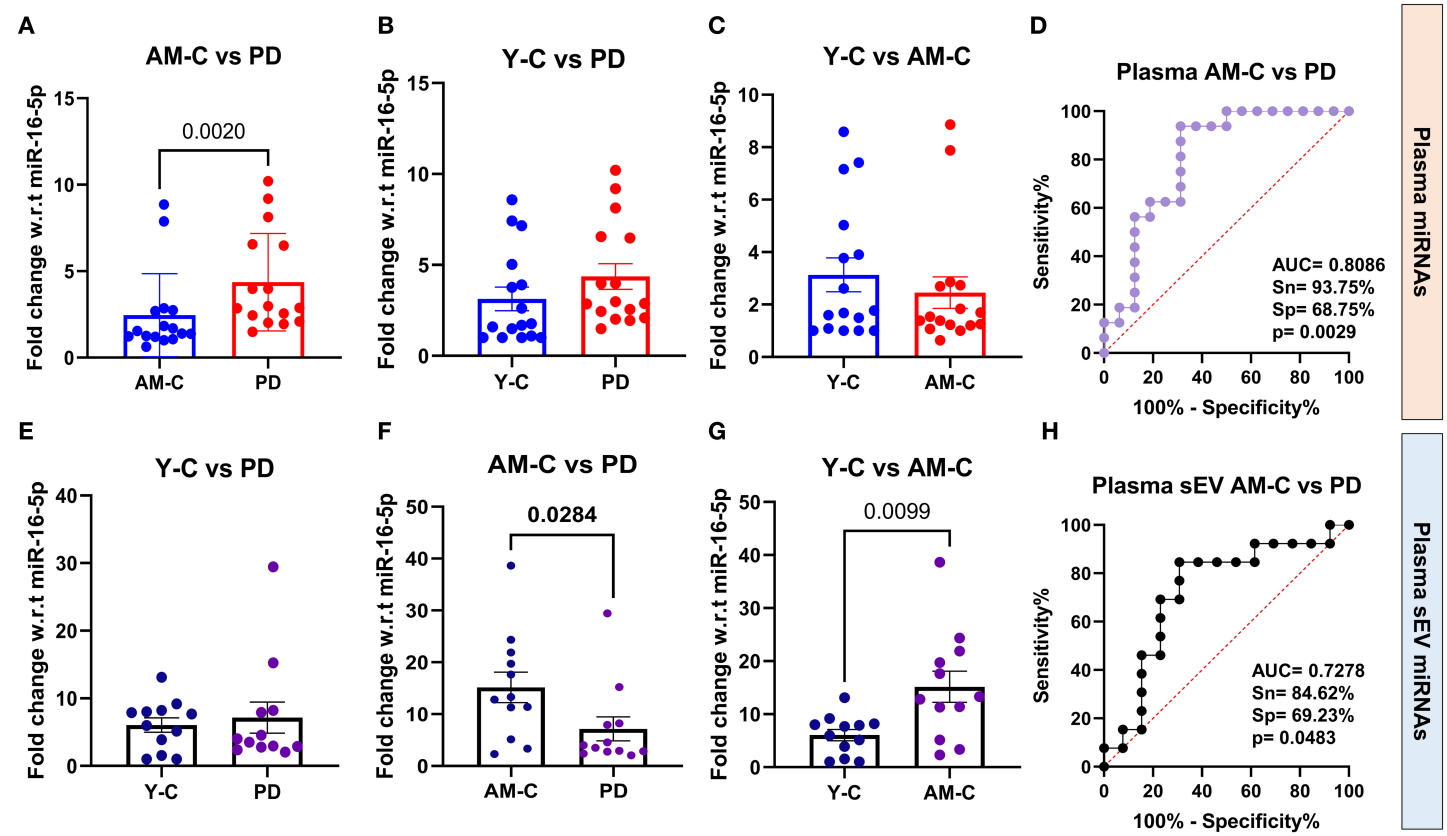
Quantitative PCR analysis and ROC analysis of differentially expressed miRNA miR-23b-3p. Comparative expression profile of miR-23b-3p in age-matched controls and PD patients **(A)** (*p* = 0.002), young controls and PD patients **(B)**, young controls and age-matched controls **(C)**, and ROC analysis between age-matched controls and PD patients **(D)** in plasma miRNA. Similarly, the comparative expression profile of miR-23b-3p in age-matched controls and PD patients **(E)**, young controls and PD patients **(F)** (*p* = 0.0284), young controls and age-matched controls **(G)** (*p* = 0.0099), and ROC analysis between age-matched controls and PD patients **(H)** in plasma-derived sEVs. Young controls (Y-C) and age-matched controls (AM-C).

Since sEVs are a part of the whole plasma, we wanted to evaluate the relative abundance of the above-selected miRNAs in both the running plasma and plasma-derived sEV of the same subject. We aim to understand if the miRNA profile in plasma is reflected the same in sEVs or is different as previously reported (Endzelinš et al., 2017; Min et al., 2019). The fold change miRNA expression is presented in Table 4 in all three experimental groups, and the differences between groups were calculated using Mann–Whitney test. Our results showed that the expression level of miR-23b-3p was “upregulated” in disease condition in PD plasma in comparison to age-matched control (*p* = 0.002; Figure 5A); however, when the same was compared with young control, we observed no significant difference (*p* = 0.0653; Figure 5B). On the contrary, we observed that the expression of miR-23b-3p was “downregulated” in the plasma-derived sEVs isolated from PD patients in comparison to age-matched controls (*p* = 0.0284; Figure 5F). Furthermore, if we compared the miRNA expression with that of the young control, there was no significant difference observed (*p* = 0.6413; Figure 5E). Moreover, no difference in expression was observed between young control and age-matched controls in plasma (*p* = 0.6413; Figure 5C); however, a significant difference was observed between the same in plasma-derived sEV fraction (*p* = 0.0099; Figure 5G). Additionally, we also looked at the correlation between plasma and plasma-derived sEV abundance and found no correlation in all three groups, viz, young control (*r* = 0.5215), age-matched control (*r* = 0.4182), and PD patient (*r* = 0.4725; Supplementary Figures 9E–G).

**Table 4:** Differential miRNA expression in fold change w.r.t. miR-16-5p.

	**miRNA expression in fold change w.r.t. miR-16-5p (mean** ±**SEM)**		
	**Young controls**	**Age-matched controls**	**PD patients**
Plasma (*n* = 16)	3.132 ± 0.647	2.454 ± 0.601	4.363 ± 0.705
Plasma-derived sEVs (*n* = 12)	6.053 ± 1.068	15.170 ± 2.951	7.161 ± 2.299

### Comparison of diagnostic efficacy between plasma and plasma-derived sEVs miR-23b-3p

We further looked into the group analysis of the miR-23b-3p expression in all three groups simultaneously; a significant upregulation of miR-23b-3p expression was observed in plasma fraction (*p* = 0.0149). However, contrasting results were observed in the plasma-derived sEV fraction, where the miRNA expression was significantly downregulated (*p* = 0.0227; Supplementary Figures 9A, B). We also performed ROC analysis to evaluate the accuracy of differentially expressed miR-23b-3p in both plasma and plasma-derived sEVs, to differentiate between the diseased and control groups (Mandrekar, 2010). The ROC analysis showed that the miR-23b-3p level in plasma had higher AUC values than the plasma sEVs, and this observation was consistent when the analysis was carried out with age-matched and young control one by one, which indicates that this miRNA abundance in plasma-derived sEVs may not be associated with the plasma abundance. Consequently, we performed the ROC curve analysis in both age-matched controls and young controls to assess the miR-23b-3p diagnostic efficacy, which revealed that the comparison with age-matched controls gave better AUC values both in plasma (AUC = 0.8086, 93.75% sensitivity, 68.75% specificity, *p* = 0.0029; Figure 5D) and plasma-derived sEVs (AUC = 0.7278, 84.62% sensitivity, 69.23% specificity, *p* = 0.0483), respectively (Figure 5H; Supplementary Figures 9C, D). This finding reflects that the expression of miR-23b-3P is contrary to plasma and plasma-derived sEV; in addition, it attests to the need for an appropriate control while assessing the diagnostic potential of any miRNA as biomarker targets.

## Discussion and conclusion

Recently, small extracellular vesicles have garnered huge attention for their potential as a minimally invasive source of diagnostic biomarkers (Kalani et al., 2014). Although, the cargo contained within these sEVs are a variety including proteins, lipids and nucleic acids (Zhang et al., 2019b). The miRNAs, a class of small non-coding RNAs, have emerged as the most studied cargo owing to their diverse function and multiple targets (Duréndez-Sáez et al., 2022). The miRNAs are released by cells in extracellular space encapsulated within the small EVs or without encapsulation in a vesicle-free form (Lee et al., 2019).

In this study, the isolated small extracellular vesicles were characterized for purity, size, and concentration utilizing TEM and NTA. We observed a characteristic lipid bilayer vesicular structure with high purity in electron micrographs of both PD patients and controls. For further validation, we performed a Western blot using antibodies against surface marker proteins such as CD9, Flotillin-1, and L1CAM as neuronal markers (Samanta et al., 2018; Ekström et al., 2022). Following the characterization and validation of isolated small extracellular vesicles (sEV), differentially expressed miRNAs were isolated and identified by small RNA sequencing using the Illumina platform. The bioinformatics analyses of sequencing data revealed that 263 miRNAs were specific to the PD group and 130 miRNAs were specific to the control group. There were 14.89% upregulated and 16.54% downregulated miRNAs where the log_2_ fold change ranged from −7.28 to 11.04. Finally, we selected four miRNA targets based on their differential expression profile in our sequencing data, which were also corroborated with the research literature. For the validation of the selected miRNA panel, we have performed qPCR using miR-16-5p as endogenous controls, which have been extensively used in qPCR studies in both plasma and sEVs (Lange et al., 2017; Reis et al., 2020). The comparative CT method (ΔΔCt Method) was used for the qRT-PCR data analysis (Bustin et al., 2009).

Unlike most neurodegenerative diseases, Parkinson's disease does not have a reliable biochemical marker as of yet (He et al., 2018). Currently, owing to the need for standardization of these techniques in a well-characterized cohort, there is ambiguity revolving around whether the sEV-derived miRNA-based assays are suitable as compared with the whole plasma-based assays. On that account, we have conducted a systematic analysis of plasma and plasma-derived miRNA in a well-characterized cohort of Parkinson's disease patients and controls (both age-matched and young control). We have shown that there is an opposite expression profile of miR-23b-3p observed both in plasma and plasma-derived sEV fractions, where the expression of miR-23b-3p is increased in plasma while the miRNA expression is decreased in plasma-derived sEV fraction. Interestingly, the reverse was observed in the lung biomarker study, where the level of miR-23b-3p was lower in plasma fraction and higher in the sEV (Chen et al., 2019). A study on miR-23b-3p in terms of Parkinson's disease reports that miR-23b-3p targets the 3′UTR of the SNCA gene, whereby it decreases the expression of SNCA gene which codes a small protein called alpha-synuclein (Norris et al., 2004; Vekrellis et al., 2011).

In addition, sEV miRNAs are thought to be more stable than free miRNAs present in plasma, due to their protective encasement in the sEV lipid bilayer, and sEVs are also considered a preferred source of miRNA biomarkers than the plasma (Endzelinš et al., 2017). However, it has also been reported that there is variability in the number of miRNA molecules packed in each sEV (Chevillet et al., 2014). Additionally, sEV isolation steps may introduce further variations in the expressions of potential miRNA biomarkers (Witwer et al., 2013), as a result of which the applicability of using sEVs or whole plasma, even for miRNA biomarker assessment in different diseases, is still challenging (Paterson et al., 2022).

In our study, we have tried to address these challenges by the standard Minimum Information for Publication of Quantitative Real-Time PCR Experiments (MIQE) recommended normalization approaches using endogenous control and the comparative CT method (ΔΔCt Method) to interpret the qRT-PCR data (Bustin et al., 2009). Furthermore, the plasma-derived sEVs were well-characterized and validated in accordance with the MISEV guidelines (Théry et al., 2018), which clearly outline the key requirements for working with sEVs prior to their downstream application. In addition, since sEV yield can be affected by factors such as venepuncture technique (Štukelj et al., 2017) and sample processing steps such as centrifugation steps, storage temperature (Bæk et al., 2016), anti-coagulants, and dietary and health status (Crewe et al., 2018), we have evaluated the relative abundance of the miRNAs in both the running plasma and plasma-derived sEV from the same sample vial, where all these confounding factors are minimized.

Although our study has a limited sample size, the validated result of small RNA sequencing results in plasma-derived sEVs and plasma for this particular miRNA (miR-23b-3p) was not a match. This could be explained due to the sequencing bias which is common in sequencing strategies based on adapter ligation resulting in the misrepresentation of transcripts (Benesova et al., 2021), whereby validation by qPCR is to be reckoned for added value (Coenye, 2021). The contrasting expression of the miRNA (miR-23b-3p) was also reported (Chen et al., 2019), where the preferred source for the differential diagnosis was concluded as plasma in their study. The reports have shown that the CNS-derived miRNAs were diluted in blood than CSF, and studying miRNA profiles in biofluids such as blood plasma or serum, which are a vast reservoir of biomolecules, gives inconsistent results (Endzelinš et al., 2017; Wang et al., 2021). miRNAs enriched in small extracellular vesicles are known to contain a reliable disease-specific miRNA profile that is not only in high abundance but also shows remarkable stability and resistance to degradation within the lipid envelope (Petrescu et al., 2019). Furthermore, sEV concentration increases in the disease condition as reported in another study (Longobardi et al., 2022), and our Western blot of sEV protein markers align with these results. Here, we do not assert better differentiation in plasma, but we would like to bring attention to the dynamic expression of this particular miRNA (miR-23b-3p). The contradictory finding of miR-23b-3p expression in plasma and plasma-derived sEVs could be attributed to myriad downstream targets of this miRNA (Hashimoto et al., 2013).

In our study, the plasma expression of miR-23b-3p was observed to be significantly decreased in age-matched control in comparison with the PD patient (*p* = 0.002), while comparing the expression in young control with PD patient showed decreased but no significance (*p* = 0.0653). Interestingly, the reverse was observed in plasma-derived sEV fraction, where miR-23b-3p expression was significantly increased in age-matched control (*p* = 0.0284) when compared with the PD group, and there was no significant difference between the young control and PD group (*p* = 0.8298). This finding also conveys the need for appropriate control while analyzing the differential expression, which is indispensable not only to miRNA studies but also to any scientific method based on experimentation and observation (Torday and Baluška, 2019). Furthermore, the plasma expression of miR-23b-3p did not differ between young control and age-matched control, but a significant difference was observed in plasma-derived sEV fraction, which can be attributed to the fact that the miRNAs have regulatory functions in aging processes (Smith-Vikos and Slack, 2012; Hatse et al., 2014). As per previous studies, the role of miR-23b-3p in PD pathogenesis has already been discussed (Su et al., 2018; Cai et al., 2021), as well as miR-23b-3p is also reported to be involved in regulating autophagy and apoptosis (Sun et al., 2018; Zhao et al., 2018), tau phosphorylation (Jiang et al., 2022), and DNA damage response (Krumeich et al., 2021).

Moreover, the differential expression of miR-23b-3p which is contrasting in plasma and plasma-derived miRNAs, as well as the differences in the young and age-matched healthy controls, needs to be thoroughly evaluated for the functional role of the miR-23b-3p. This disparity implies a subtle regulation of miR-23b-3p, and further explanatory studies are recommended to understand the role of miR-23b-3p not only in terms of disease pathophysiology but also in healthy physiological system.

## Data availability statement

The original contributions presented in the study are included in the article/Supplementary material, further inquiries can be directed to the corresponding author.

## Ethics statement

The studies involving human participants were reviewed and approved by Institutional Ethics Committee, All Institute of Medical Sciences, New Delhi, India (Ref. No.: IECPG-766/30.11.2022). The patients/participants provided their written informed consent to participate in this study.

## Author contributions

SK: conceptualization and design of the study. SRai, PB, RS, SRas, SY, and SK: acquisition and analysis of data. SRai, PB, RS, KR, VS, PG, and SK: drafting the text or preparing the figures. SK, FN, RR, SY, HP, KI, GM, BV, NR, and TR: initial revision and proofreading of the manuscript. All authors contributed to the article and approved the submitted version.
