# Supplementary material for: Circulating plasma miR-23b-3p as a biomarker target for idiopathic Parkinson's disease: comparison with small extracellular vesicle miRNA
Source: Front Neurosci. 2023 Nov 15;17:1174951. doi: 10.3389/fnins.2023.1174951 (PMC10684698; doi:10.3389/fnins.2023.1174951)
Supplement: Supplementary file 1 [file Data_Sheet_1.docx]

**Supplementary Table S1: Demographic details of each subject.**

| **S. No.** | **Age (in years)** | | | **Gender** | | | **Modified-HY Score in PD Patients** |
| --- | --- | --- | --- | --- | --- | --- | --- |
|  | **Young Controls** | **Age-Matched Controls** | **PD Patients** | **Young Controls** | **Age-Matched Controls** | **PD Patients** |  |
|  | 29 | 56 | 63 | M | M | M | 2.5 |
|  | 27 | 54 | 64 | M | F | F | 3 |
|  | 27 | 85 | 76 | M | M | M | 3 |
|  | 26 | 49 | 61 | M | M | M | 2.5 |
|  | 21 | 55 | 78 | M | M | M | 2.5 |
|  | 25 | 45 | 38 | M | M | M | 2 |
|  | 25 | 45 | 65 | M | M | F | 2.5 |
|  | 28 | 45 | 37 | M | M | M | 1.5 |
|  | 30 | 52 | 50 | M | M | M | 2.5 |
|  | 24 | 52 | 33 | F | F | M | 1.5 |
|  | 26 | 50 | 52 | M | M | M | 2.5 |
|  | 25 | 68 | 69 | M | F | M | 2 |
|  | 23 | 82 | 56 | M | M | M | 3 |
|  | 30 | 56 | 56 | M | F | F | 3 |
|  | 23 | 45 | 37 | M | M | M | 2.5 |
|  | 26 | 44 | 51 | F | M | M | 2.5 |


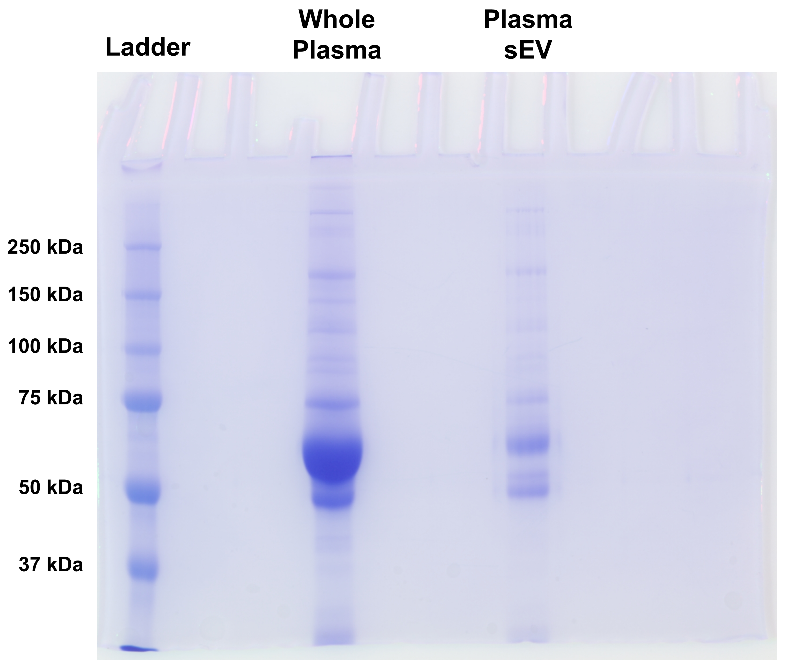


**Supplementary Figure S2: Coomassie-stained gel image showing Apolipoprotein and Albumin contamination in whole plasma versus plasma-derived sEV fraction.**

**Supplementary Table S3: The size distribution of plasma-derived sEVs.**

|  | **Young Controls** | **Age-Matched Controls** | **PD Patients** |
| --- | --- | --- | --- |
| **sEV Size in nm (Mean ±SD)** | 80.15 ± 28.20 | 89.18 ± 14.59 | 92.33 ± 13.08 |


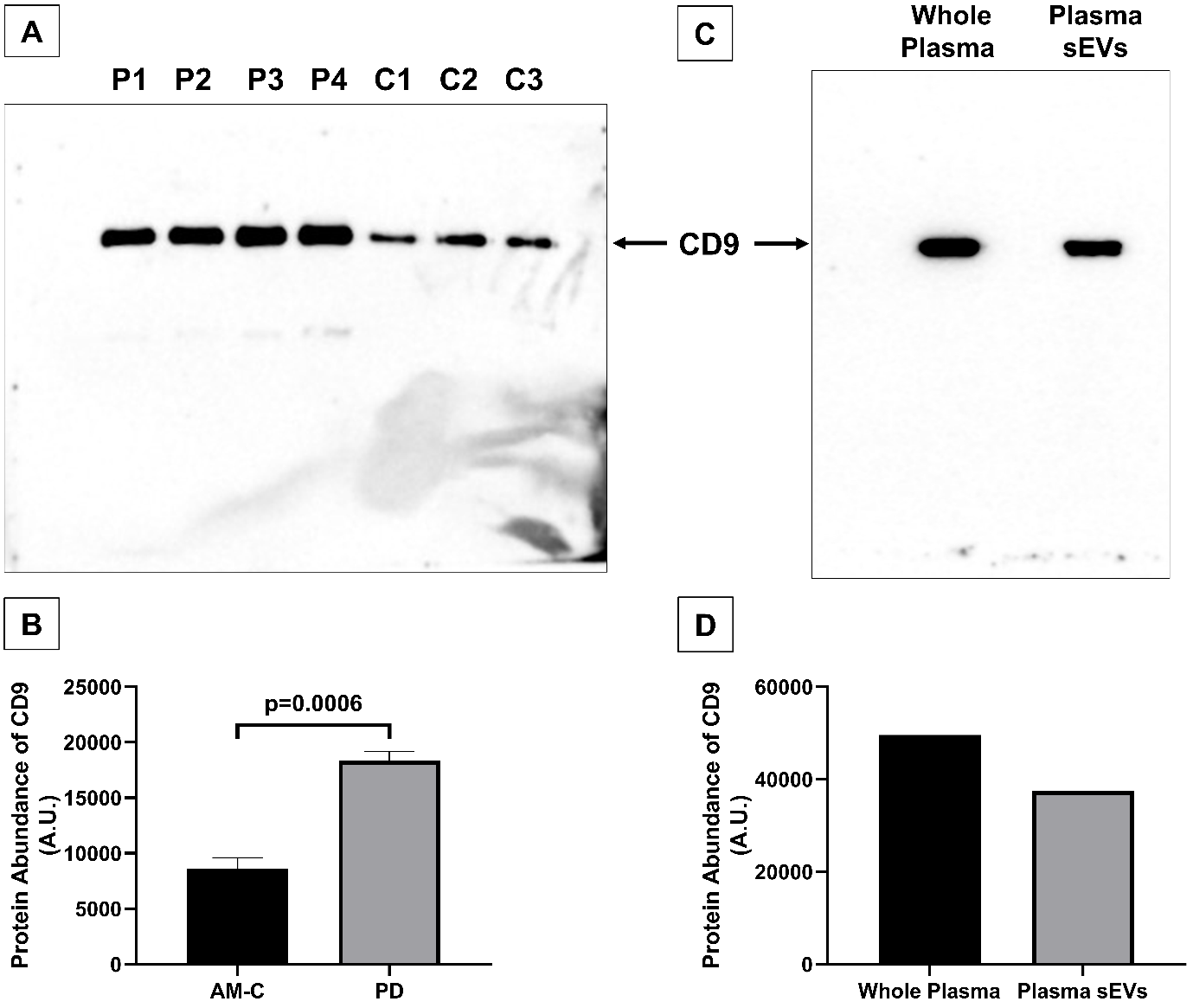


**Supplementary Figure S4: Expression profile of CD9 in PD patients (P1-P4) and age-matched healthy controls (C1-C3).** (A) Western blot of anti-CD9 with equal sample volume (5µl) in PD and age-matched healthy controls, and its densitometric analysis (p=0.0004) (B). (C) Western blot of whole plasma and plasma-derived sEVs and its densitometric analysis (D) that shows 75-80% extraction of sEVs from whole plasma. All graphs are presented with Mean ± SEM.


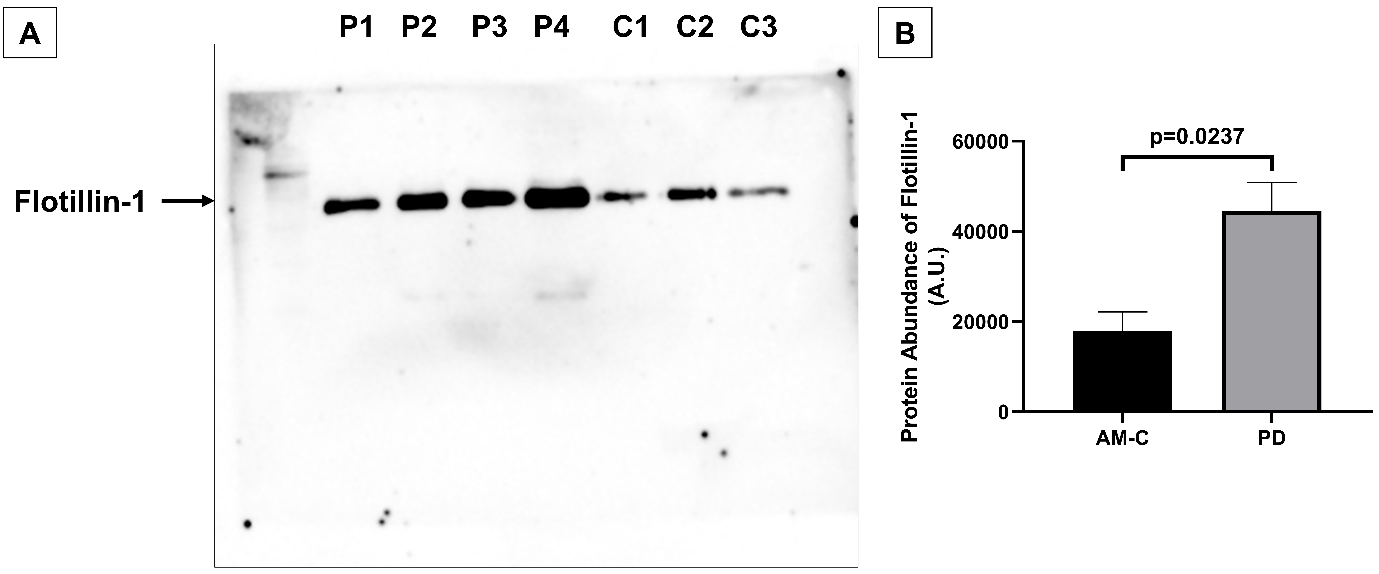


**Supplementary Figure S5: Expression profile of Flotillin-1 in PD patients (P1-P4) and age-matched healthy controls (C1-C3).** (A) Western blot of anti-Flotillin-1 with equal sample volume (5µl) in PD and age-matched healthy controls, and its densitometric analysis (p=0.0237) (B). All graphs are presented with Mean ± SEM.


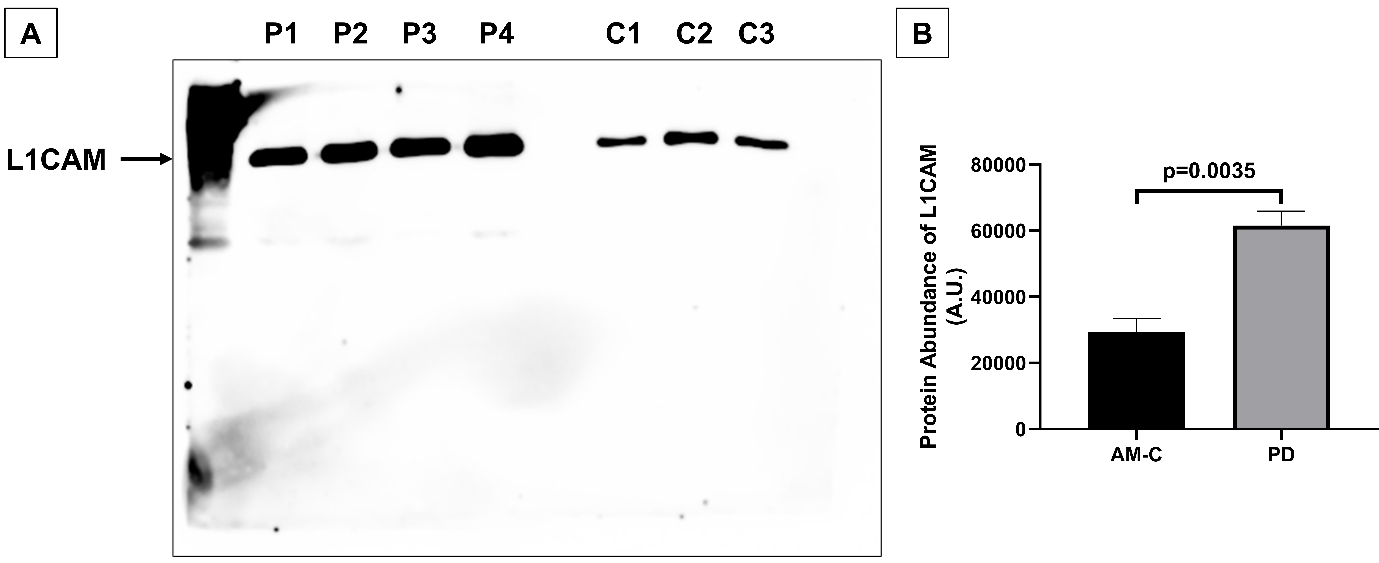


**Supplementary Figure S6: Expression profile of L1CAM in PD patients (P1-P4) and age-matched healthy controls (C1-C3).** (A) Western blot of anti-L1CAM with equal sample volume (5µl) in PD and age-matched healthy controls, and its densitometric analysis (p=0.0035) (B). All graphs are presented with Mean ± SEM.


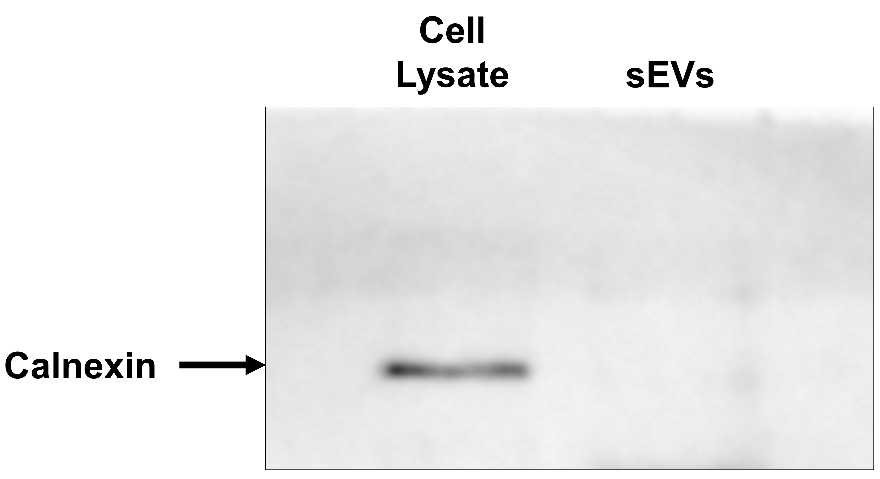


**Supplementary Figure S7: Western blot of anti-Calnexin in sEVs and Cell Lysates.** Calnexin was used to detect non-EV contamination and is an sEV-negative protein marker. CL: cell lysate.


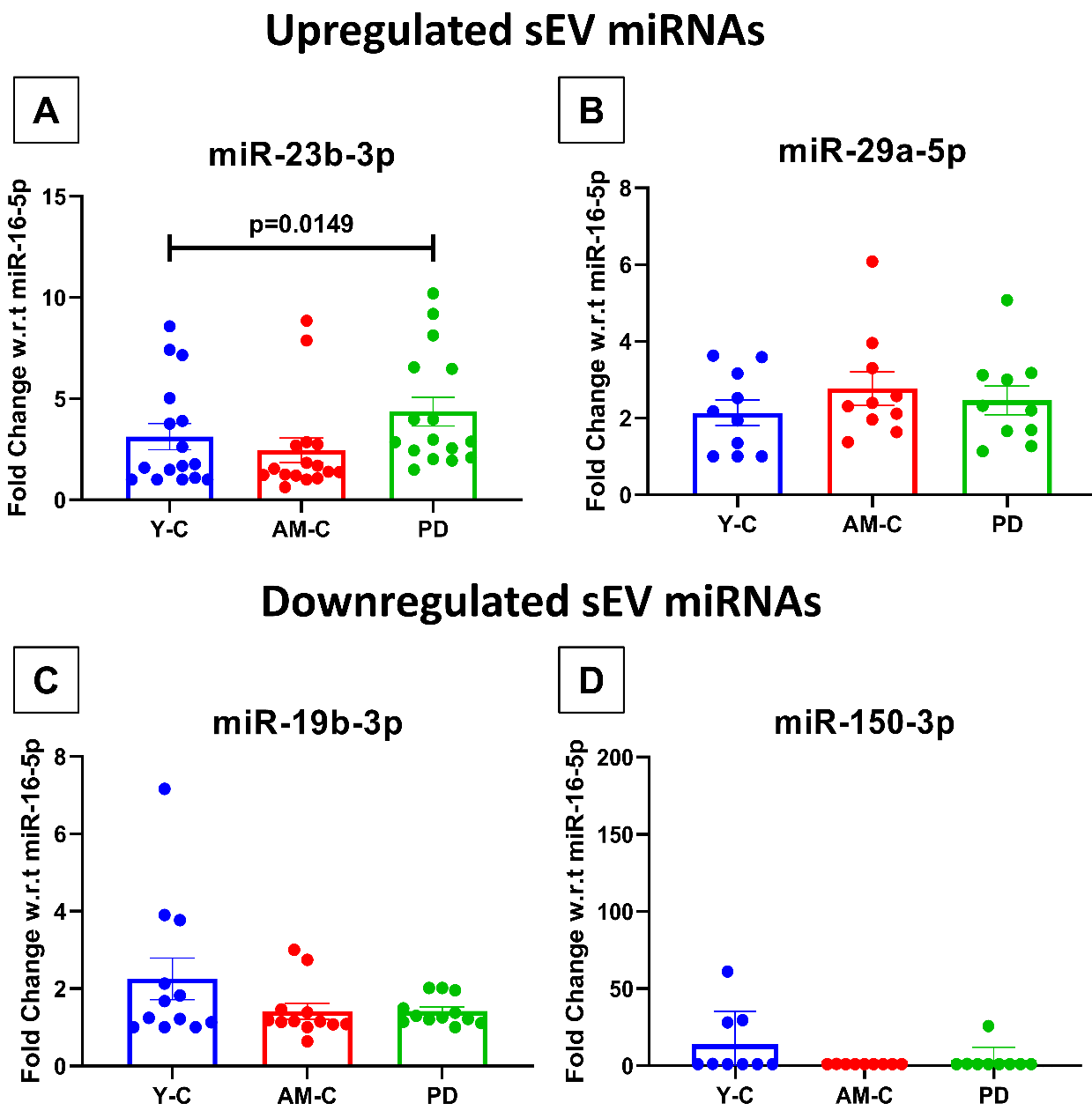


**Supplementary Figure S8: Quantitative PCR analysis of differentially expressed sEV miRNAs.** Comparative expressions of upregulated sEV miRNAs miR23b-3p (A) and miR-29a-5p (B), and downregulated sEV miRNAs miR-19b-3p (C) and miR-150-3p (D) in young controls, age-matched controls & PD patients.


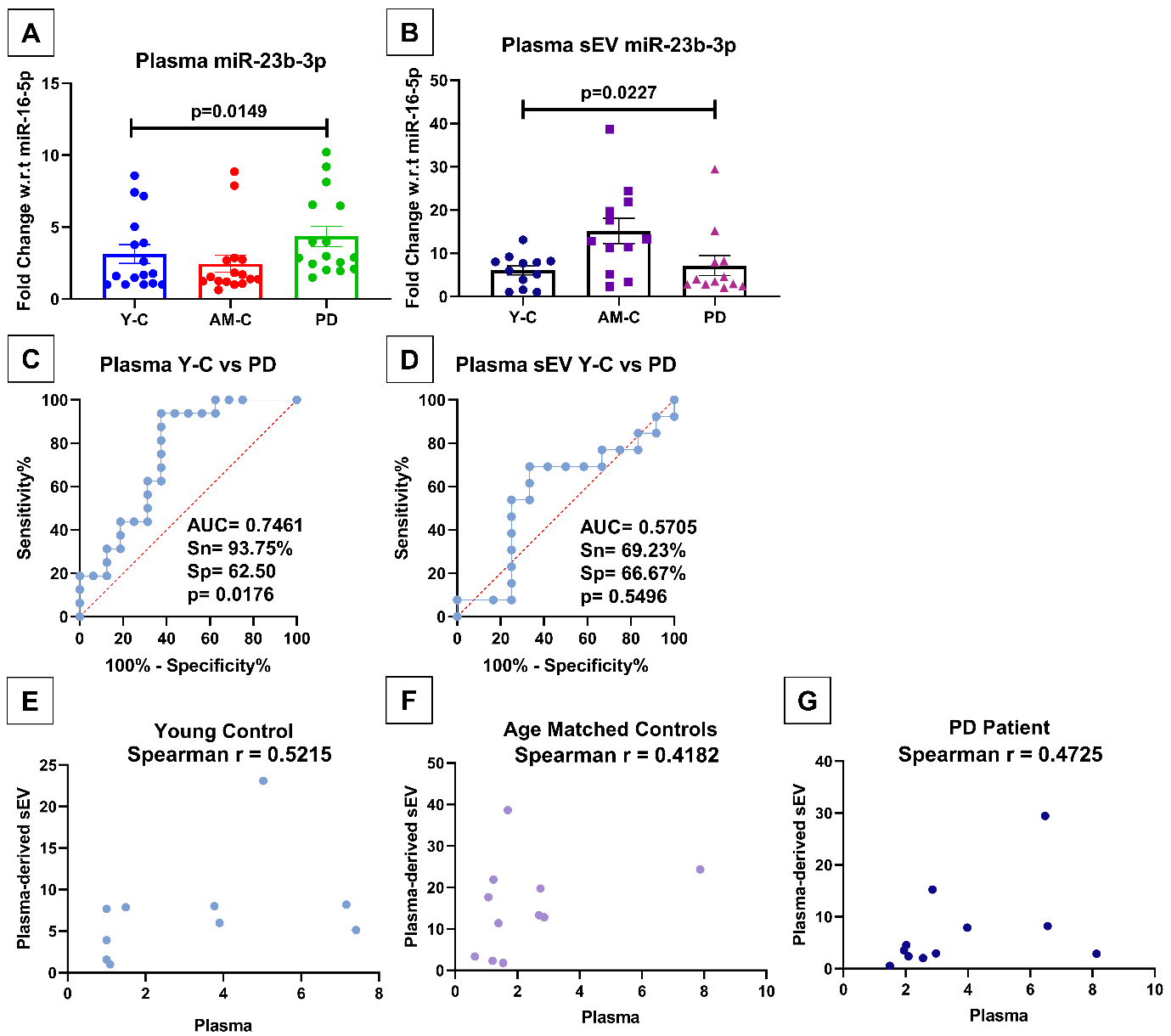


**Supplementary Figure S9: Comparative qPCR analysis, ROC analysis, and Correlation of differentially expressed miR-23b-3p.** Comparative expression profile of plasma miR-23b-3p (A), and plasma-derived sEV miR-23b-3p (B) in young controls, age-matched controls & PD patients. ROC curve analysis of young controls vs PD of plasma miRNA (C) and plasma-derived sEVs miRNA (D). Correlation analysis (Spearmen r) between plasma miRNA and plasma-derived sEV miRNA in young controls (E), age-matched controls (F), and PD patients (G).
